# Methane-powered sea spiders: Diverse, epibiotic methanotrophs serve as a source of nutrition for deep-sea methane seep *Sericosura*

**DOI:** 10.1073/pnas.2501422122

**Published:** 2025-06-16

**Authors:** Bianca Dal Bó, Yongzhao Guo, Magdalena J. Mayr, Olivia S. Pereira, Lisa A. Levin, Victoria J. Orphan, Shana K. Goffredi

**Affiliations:** ^a^Department of Biology, Occidental College, Los Angeles, CA 90041; ^b^Division of Geological and Planetary Sciences, California Institute of Technology, Pasadena, CA 91125; ^c^Division of Integrated Oceanography, Scripps Institution of Oceanography, University of California San Diego, San Diego, CA 92121

**Keywords:** symbiosis, methane, sea spider, deep-sea seeps, methanotroph

## Abstract

Methane seeps harbor uncharacterized animal–microbe symbioses with unique nutritional strategies. Three undescribed sea spider species (family Ammotheidae; genus *Sericosura*) endemic to methane seeps were found along the eastern Pacific margin, from California to Alaska, hosting diverse methane- and methanol-oxidizing bacteria on their exoskeleton. δ^13^C tissue isotope values of in situ specimens corroborated methane assimilation (−45‰, on average). Live animal incubations with ^13^C-labeled methane and methanol, followed by nanoscale secondary ion mass spectrometry, confirmed that carbon derived from both compounds was actively incorporated into the tissues within five days. Methano- and methylotrophs of the bacterial families Methylomonadaceae, Methylophagaceae and Methylophilaceae were abundant, based on environmental metagenomics and 16S rRNA sequencing, and fluorescence and electron microscopy confirmed dense epibiont aggregations on the sea spider exoskeleton. Egg sacs carried by the males hosted identical microbes suggesting vertical transmission. We propose that these sea spiders farm and feed on methanotrophic and methylotrophic bacteria, expanding the realm of animals known to harness C1 compounds as a carbon source. These findings advance our understanding of the biology of an understudied animal lineage, unlocking some of the unique nutritional links between the microbial and faunal food webs in the oceans.

## Significance

This study uncovers a unique partnership between sea spiders of the genus *Sericosura* and epibiotic methane-oxidizing bacteria. These spiders, found at methane seeps along the Pacific margin, host diverse methylotrophic bacteria on their exoskeletons, which they farm and consume. Evidence from tissue isotopic analysis, microbiome sequencing, and live-animal incubations followed by ^13^C-methane isotope probing confirms active incorporation of methane-derived carbon into spider tissues. This research highlights a previously unknown interaction between an animal lineage and chemically fueled microbes, introducing another symbiotic pathway for direct microbial transfer of methane carbon into animal biomass in the deep sea.

Methane seeps are deep-sea ecosystems where hydrocarbons emerge from the seabed to support abundant animal communities that flourish by associating with microbial communities. Bacteria and archaea are uniquely poised to gain energy directly from C1 compounds, and microbes capable of oxidizing methane and methanol are therefore prime partners for symbiotic relationships in these environments. Consequently, diverse animals rely directly on methano- and methylotrophic bacteria in methane seeps to produce organic carbon without sunlight. For example, deep-sea sponges, annelids and mytilid mussels interact with members of the methane-oxidizing Methylomonadaceae (1–7). Methanol-oxidizing Methylophagaceae also inhabit the gills of the mussels where they utilize methanol generated during methane-oxidation by the primary Methylomonadaceae symbionts (7–9). In these symbioses, chemosynthetic microbes benefit the host nutritionally, presumably in exchange for increased access to the compounds required for symbiont metabolism (10). Because they are difficult to access, methane seeps are currently understudied, but evidence accumulated in the last decade suggests that animal–microbe interactions involving methane are likely more prevalent than currently known, perhaps harboring novel modes of carbon transfer through the food web.

The largely unexplored eastern Pacific margin hosts numerous methane seeps with abundant and often distinct animal communities (11). Off of southern California, the Del Mar seep discovered in 2012, at 1,018 m depth, hosts over 60 animal species in unique microhabitats, affirming the high biodiversity of relatively small regions of the seafloor (12, 13). While most of these animals use predation or filter feeding to grow, others exhibit more complex interactions with microbial communities, and enter into nutritional relationships with symbionts of diverse metabolic capabilities. Stable isotope measurements in animal tissue [measured as δ^13^C values (14, 15)] have already provided evidence that much of the Del Mar seep macrofauna, especially when associated with authigenic carbonates, depends upon chemosynthetic production (12). Marine chelicerates are of particular interest, as they display tissue δ^13^C values that are lower than expected for their typical mode of feeding [δ^13^C of −31‰ (12)]. These animals, commonly referred to as sea spiders (class Pycnogonida), are a widespread yet understudied epibenthic invertebrate family. They typically feed on slow, soft-bodied invertebrates with their piercing-sucking mouthparts (16, 17). Interestingly, the genus *Sericosura* (family Ammotheidae) has only been described from chemosynthetic habitats, and in very high numbers (18–20). We predict that the “unusual” chelicerate sampled from the Del Mar seep (12) belongs to the genus *Sericosura* since this genus has been the only sea spider lineage detected so far during several expeditions to this site. Importantly, early observations failed to identify prey from *Sericosura* stomach contents (18). Several lines of evidence therefore suggest that members of this genus use a nutritional strategy different from that of other pycnogonids, and one that has not yet been characterized.

To test this hypothesis, we examined whether *Sericosura* sea spiders from methane seeps rely on methano- or methylotrophic bacteria, and characterized the specific nature and mechanism of this possible interaction. Three undescribed species of sea spider (genus *Sericosura;* family Ammotheidae) were collected from methane seeps along North American coastlines. Specimens showed strongly depleted ^13^C in their tissues and active incorporation of both methane- and methanol-derived carbon. Microbiome analysis revealed diverse methane- and methanol-oxidizing (MMOx) bacteria attached to the exoskeleton of all three species. These epibionts include methanotrophic members of the family Methylomonadaceae, as well as the methylotrophic Methylophagaceae and Methylophilaceae families. Together, evidence points to a system where the host grazes on symbionts for carbon acquisition. The association of three MMOx bacterial families with the *Sericosura* sea spiders, and their role for animal nutrition has implications from both a host and symbiont perspective, as it expands i) the diversity of methylotrophic bacteria associated with a single animal genus, ii) the breadth of invertebrate phyla hosting these groups and iii) the range of known hosts that are nutritionally reliant on methane-derived carbon.

## Results

### *Sericosura* Sea Spiders Are Common at Methane Seeps.

**Fig. 1.**
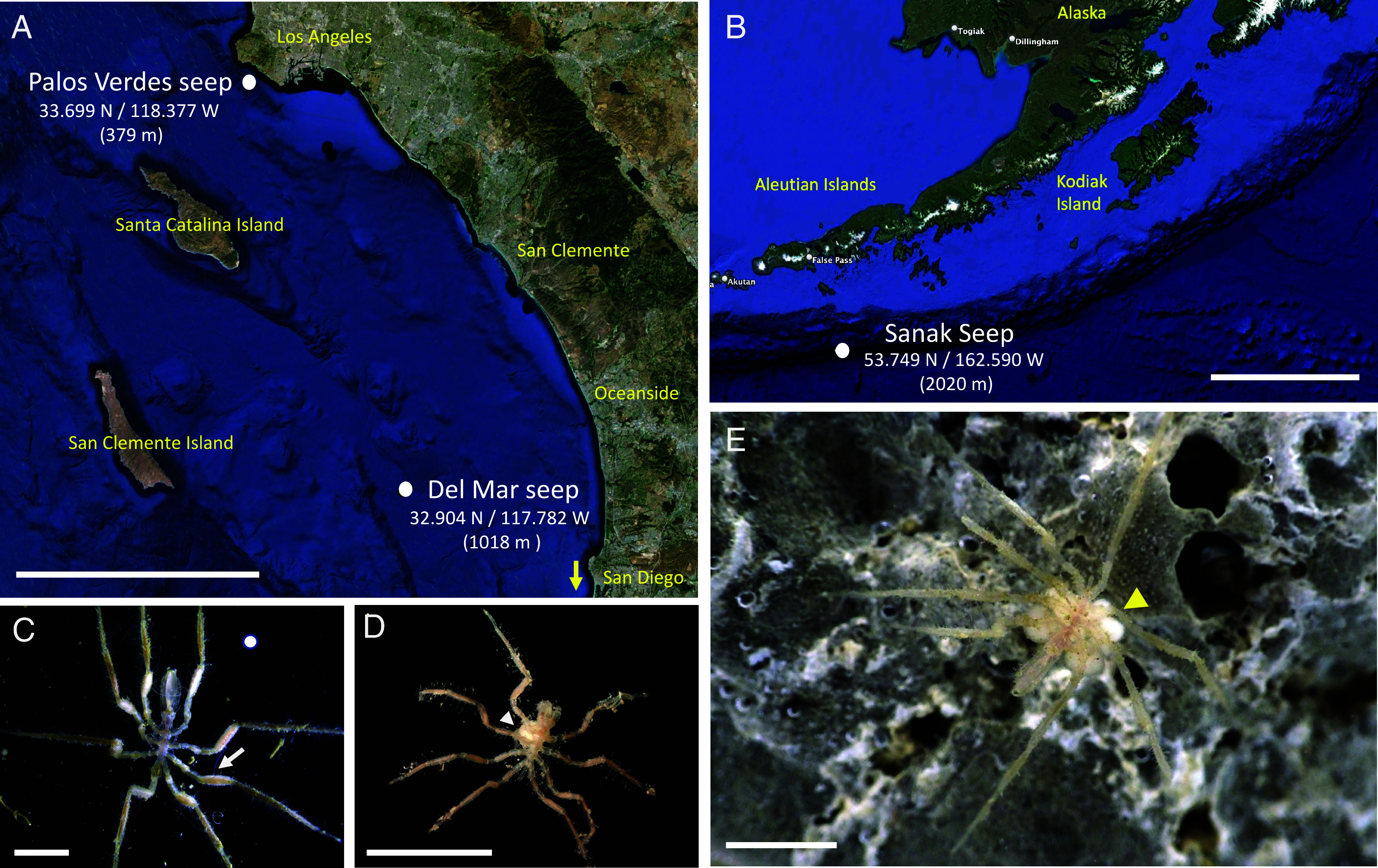
*Sericosura* sea spiders examined in this study. (*A*) Map of the Southern California seep locations. (*B*) Map of the Sanak Seep site off of the Aleutian Islands. (*C*) A female from the Del Mar seep clearly showing swollen femora with eggs (arrow). (*D*) A male from the Sanak seep clearly showing egg brood (arrowhead). (*E*) An inconspicuous *Sericosura* male specimen walking on a carbonate rock at the Del Mar seep (the arrowhead denotes egg brood). (Scale bars: *A*, 80 km. *B*, 300 km. and *C*–*E*, 2 mm.) Photo credits: *C* and *E*, Bianca Dal Bó; *D*, Greg Rouse (SIO).

*Sericosura* sea spiders were regularly associated with authigenic carbonates in areas of active seepage (Fig. 1), and were not observed or recovered from the seep periphery ~25 m away from the seep. Sequencing of the cytochrome oxidase I (COI) gene confirmed that specimens from all three sites (Del Mar, Palos Verdes and Sanak seeps) each represent new, undescribed species of *Sericosura*, 91 to 93% similar to other described species, including *S. venticola* and *S. verenae*, reported from the Juan de Fuca hydrothermal vents. These new *Sericosura* species are currently under description. The largest collection of sea spiders occurred during the 2023 expedition at the Del Mar seep, with a total of 33 individuals (17 males, 12 females, and 4 juveniles; females had enlarged femora (Fig. 1*C*) with eggs stored prior to mating (16) and males had brood present (Fig. 1 *D* and *E*).

### The *Sericosura* Microbiome Is Dominated by Diverse Methylotrophic Bacteria.

**Fig. 2.**
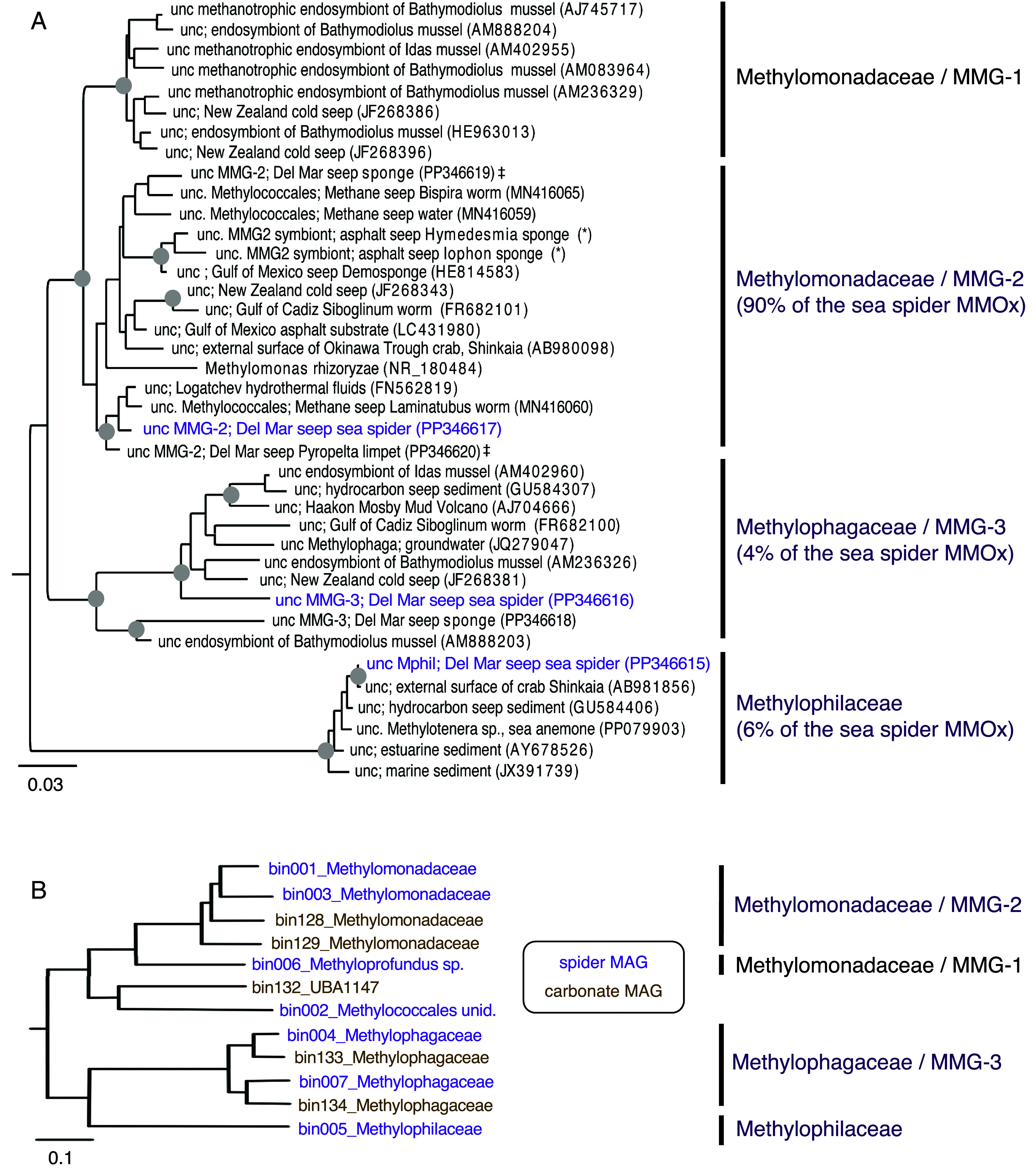
Phylogenetic relationships among the methanotrophic and methylotrophic bacterial families associated with *Sericosura* sea spiders. (*A*) 16S rRNA gene sequences. Purple denotes taxa found in this study in the sea spider microbiome. Symbol (‡) indicates two other Del Mar animals also associated with MMG-2 bacteria in this study. *Methylosphaera hansonii* (U67929) was used as the outgroup. Circles at nodes indicate bootstrap support (>0.7), aligned using Geneious Prime 2021.2.2 (neighbor-joining, Tamura-Nei model). Additional sequences from environmental and cultured relatives were obtained from GenBank. The asterisk (*) indicates sequences provided by Maxim Rubin-Blum (Israel Oceanographic and Limnological Research). (*B*) Phylogenetic relationships among the Methylococcales, Methylophagaceae and Methylophilaceae MAGs of sea-spiders, compared to those recovered from carbonates. All nodes were supported with bootstrap values of 1.

The microbial community of whole *Sericosura* specimens was dominated by methanotrophic and methylotrophic bacteria, according to 16S rRNA sequencing (short amplicon and longer-read diagnostic) and environmental metagenomics. Community profiling showed that Methylomonadaceae (Methylococcales) comprised 22 to 61% of the microbiome of all three *Sericosura* species from the three sites (*SI Appendix*, Fig. S1). The egg sacs carried by the Del Mar seep *Sericosura* males also hosted Methylomonadaceae (19 to 29% relative abundance; *SI Appendix*, Figs. S1 and S2*B*). Of the top 10 amplicon sequence variants (ASVs) responsible for the uniqueness of the Del Mar *Sericosura* microbiome, six belonged to the Marine Methylotrophic Group-2 (MMG-2) of the Methylomonadaceae (4) [similarity percentage (SIMPER) analysis; *SI Appendix*, Fig. S2*A*]. Three identical ASVs were recovered from all three host species (*SI Appendix*, Fig. S2*A*). A longer 16S rRNA gene sequence from the Del Mar specimens corresponded to the dominant ASV from the 16S rRNA barcoding analysis (*SI Appendix*, Fig. S2*B*), and was most closely related to the methane-oxidizing MMG-2 symbiont of *Laminatubus* serpulids from a Costa Rican methane seep (6) (Fig. 2*A*; 96% similarity). This sea spider-serpulid MMG-2 lineage was distinct from those recovered from Del Mar seep sponges and from those commonly recovered from bathymodioline mussels, known as the Marine Methylotrophic Group-1 (9, 21) (Fig. 2*A*). Two MMG-2 metagenome-assembled genomes (MAGs) recovered from the Del Mar sea spiders had an average nucleotide identity (ANI) of 76% (Fig. 2*B* and *SI Appendix*, Table S1), and encoded genes associated with methanotrophic and methylotrophic metabolism, including particulate methane monooxygenase (pmoCAB) and methanol dehydrogenase genes (xoxF; *SI Appendix*, Figs. S3 and S4). Finally, members of the Methylomonadaceae were recovered from carbonate surfaces from which the Del Mar seep sea spiders were collected, yet they appeared distinct from the sea spider-associated MMG-2 (Fig. 2*B* and *SI Appendix*, Fig. S2*B*). MAGs of the carbonate-associated Methylomonadaceae had maximum ANI values of 77% compared to similar lineages on the sea spiders (Fig. 2*B*).

Two additional MMOx bacterial families, the Methylophagaceae (Nitrosococcales) and Methylophilaceae (Burkholderiales), were also recovered from all three undescribed *Sericosura* species, albeit at much lower abundances than the Methylomonadaceae (average 1 to 2% of the total community, based on 16S rRNA amplicon sequencing; *SI Appendix*, Fig. S1). The egg sacs carried by the Del Mar seep *Sericosura* males also harbored both methylotrophic families; Methylophagaceae up to 1.5% and Methylophilaceae up to 5.5%; *SI Appendix*, Fig. S1). Within these minor community members, two ASVs represented 90% of the Methylophagaceae family for the Del Mar sea spiders and were shared among the three species (*SI Appendix*, Fig. S2*A*). This Del Mar sea spider-associated group was affiliated with the Methylophagaceae Marine Methylotrophic Group-3 (MMG-3) (4), a group previously described from mytilid mussels *Idas* and *Bathymodiolus* (8) (Fig. 2*A*). Similarly, while three Methylophilaceae ASVs were recovered from the Del Mar sea spider population (*SI Appendix*, Fig. S2*A*), a single dominant ASV represented 80+% of the Methylophilaceae amplicons. The dominant sea spider-associated Methylophilaceae was 97% similar to an epibiont recovered from the hairy crab *Shinkaia* (22), as well as other free-living bacteria from reducing sediments including methane seeps (23) (Fig. 2*A*). Like the MMG-2 group, whole genome sequencing produced two primary MMG-3 MAGs from the Del Mar sea spider-associated bacterial community (with 78% nucleotide-level genomic similarity between them) and a single Methylophilaceae MAG (Fig. 2*B* and *SI Appendix*, Table S1). Both the Methylophilaceae and the MMG-3 MAGs contained methanol dehydrogenase genes consistent with other members of these groups (*SI Appendix*, Figs. S3 and S4). Members of the Methylophagaceae MMG-3 group were also recovered from Del Mar seep carbonates (Fig. 2*B* and *SI Appendix*, Fig. S2*B*) and carbonate-associated metagenomic-assembled genomes had maximum ANI values of 82% to similar lineages recovered from the sea spiders (Fig. 2*B*). By contrast, no Methylophilaceae were recovered from Del Mar seep carbonate surfaces, either via 16S rRNA amplicon sequencing or environmental metagenomics.

### Fluorescence and Electron Microscopy Revealed the Presence of Putative MMOx Epibionts on *Sericosura*.

**Fig. 3.**
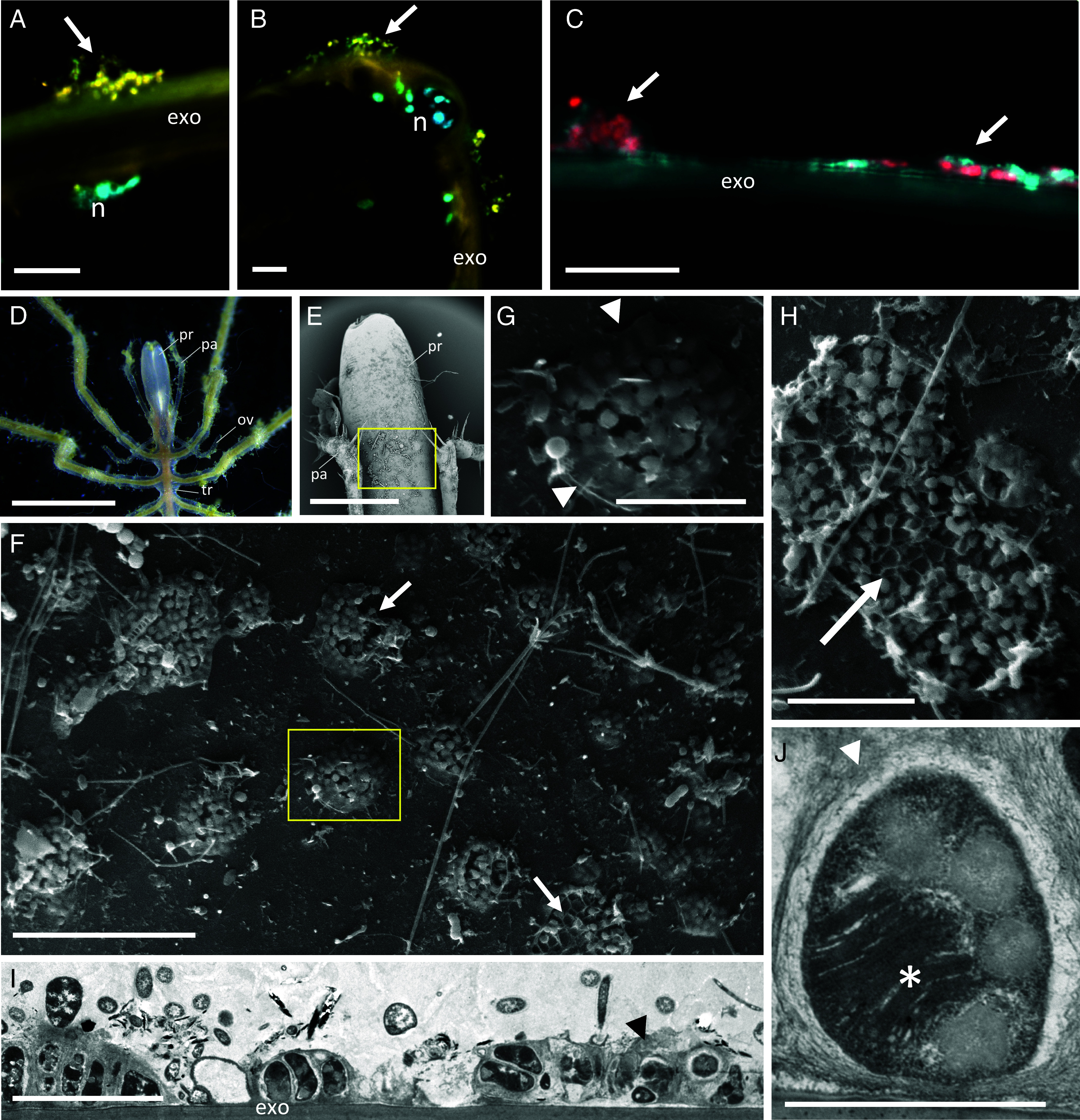
Bacteria associated with *Sericosura* sea spiders. (*A* and *B*) FISH microscopy showing bacterial aggregations (arrows; in yellow) with a probe targeting the methanotrophic Methylomonadaceae (MTC850 labeled with Cy3) and sea spider nuclei stained with DAPI (in cyan), amid high autofluorescence of the exoskeleton (exo). n, nucleus. (*C*) FISH microscopy of bacterial aggregations (arrow; in red) using a probe designed to target the Methylococcales more broadly (MTMC701 labeled with Cy5), counterstained with DAPI (cyan). There was no qualitative difference between results with probes MTC850 and MTMC701 (Fig. 3*A* vs. 3*C*). (*D*) Sea spider, showing proboscis (pr), flanked by a pair of palps (pa). ov, ovigers; tr, trunk. (*E*) SEM of the dorsal surface of the proboscis. The box denotes the region where numerous bacterial aggregates (arrows) were observed, and shown in *F* and *G*. (*F*) SEM revealed numerous, uniformly spaced, volcano-like bacterial aggregations. The box denotes the region in *G*. (*G*) SEM close-up of cells clustered together in a single aggregate, surrounded by an apparent EPS layer (arrowheads). (*H*) SEM of a single aggregate of uniform ovoid cells, some of which appear missing or disturbed (arrow). (*I*) TEM of aggregations of the putative MMOx bacteria, attached to the surface of the exoskeleton, surrounded by and attached to other bacterial community members. (*J*) TEM of a single bacterium, putatively identified as a methanotroph based on the dense stacked intracytoplasmic membranes (*). In *I* and *J*, the apparent EPS layer is denoted by arrowheads. (Scale bars: *A*–*C*, 10 μm. *D*, 2 mm. *E*, 500 μm. *F*, 20 μm. *G*, 5 μm. *H*, 10 μm. *I*, 5 μm. and *J*, 1 μm.)

The identity of methanotrophic Methylomonadaceae bacteria in cell aggregations on the external surfaces of *Sericosura* was confirmed using fluorescence in situ hybridization (FISH) probes targeting the family specifically [MTC850 (4)] and Methylococcales order more broadly [MTMC701 (4); Fig. 3 *A*–*C*]. Attempts to visualize minor members of the MMOx community, including the Methylophilaceae [FISH probe MET-1217 (24)] and the Methylophagacea MMG-3 group [FISH probe MPH732 (4)] were unsuccessful. This was likely due to the much lower abundances of these bacterial groups (*SI Appendix*, Fig. S1), and the substantial autofluorescence of the exoskeleton, masking a weaker signal from bacterial cells. Notably, we did not observe bacteria associated with any internal structures of the sea spiders with FISH microscopy. Scanning electron microscopy (SEM) identified numerous ovoid cells, ~1 µm in length, clustered in uniformly spaced (13 ± 4 µm), volcano-like aggregations, surrounded by an extracellular polymeric substance (EPS; Fig. 3 *D*–*G*). Some of these aggregations were intact, while others appeared disturbed (Fig. 3*H*). Transmission electron microscopy (TEM) additionally showed that the aggregated bacteria contained stacked intracytoplasmic membranes, characteristic of methanotrophs (25) (Fig. 3 *I* and *J*). TEM also confirmed the distinct surrounding EPS layer (Fig. 3 *I* and *J*). FISH and TEM of the egg sacs showed a diverse bacterial community, including the Methylomonadaceae, embedded within the external hardened mucus, but not in the egg interior (*SI Appendix*, Fig. S5).

### Isotopic Evidence of Active Methane and Methanol Incorporation by *Sericosura* Sea Spiders.

**Fig. 4.**
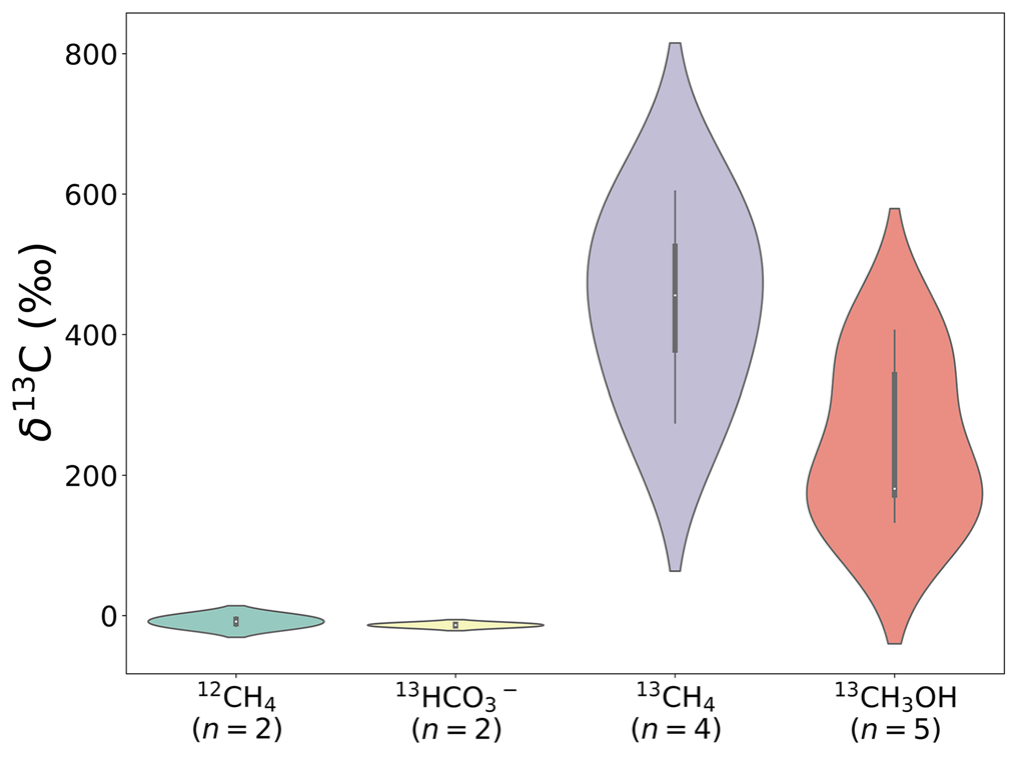
Violin plot of tissue carbon isotope values (δ^13^C‰) for experimental *Sericosura* sea spiders from the Del Mar seep, following ^13^C enrichment experiments. Live animals were incubated on-board ship, with unlabeled methane (^12^CH_4_), ^13^C-labeled bicarbonate (^13^HCO3), ^13^C-labeled methane (^13^CH_4_) or ^13^C-labeled methanol (^13^CH_3_OH) for 108 to 181 h. Sample sizes are noted. For one sea spider in a separate incubation with ^13^CH_4_ (161 h), the tissue δ^13^C value reached 1,248‰. This specimen was not included in the plot due to lack of a paired control. In the presence of both ^13^C-labeled substrates, egg cases from brooding males showed slight ^13^C enrichment (δ^13^C = 9 to 109‰, n = 1 to 2).

**Fig. 5.**
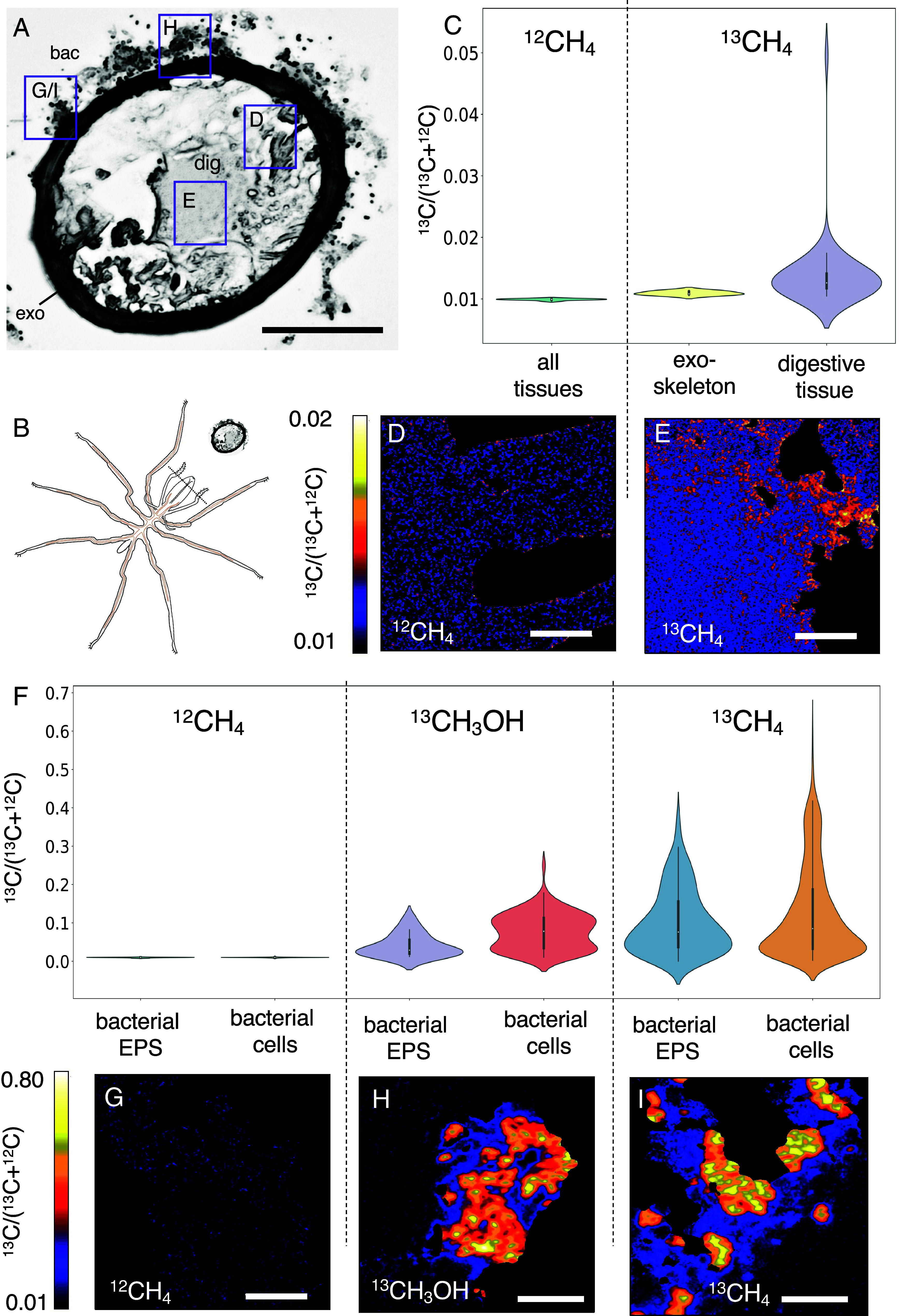
nanoSIMS analysis, following ^13^C enrichment experiments, confirms incorporation of methane- and methanol-derived carbon by sea spider epibionts and the host animal. (*A*) Transverse-section through the palp showing digestive diverticula. Boxes denote corresponding regions of nanoSIMS analysis shown in other panels. dig, digestive diverticula. exo, exoskeleton. bac, bacteria. (*B*) Illustration showing angle of proboscis cross-section in panel *A*. (*C*) Violin plot of nanoSIMS data, showing ^13^C enrichment levels in sea spider digestive tissue and exoskeleton when exposed to ^13^C-methane, versus similar regions of interest (all tissues) when exposed to ^12^C-methane. (*D*) nanoSIMS image of sea spider tissues after ^12^CH_4_ incubation, showing no enrichment in ^13^C. (*E*) nanoSIMS image revealing ^13^C enrichment of sea spider digestive tissue after ^13^CH_4_ incubation. (*F*) Violin plot of nanoSIMS data, showing ^13^C enrichment levels in sea spider-associated bacterial epibiont cells and EPS layer, when exposed to ^13^C-methane or ^13^C-methanol, versus similar regions of interest when exposed to ^12^C-methane. (*G*) nanoSIMS image of MMOx epibionts after ^12^CH_4_ incubation, showing no enrichment in ^13^C. (*H*) nanoSIMS image of ^13^C enrichment of MMOx epibionts, after ^13^CH_3_OH incubation. (*I*) nanoSIMS image of ^13^C enrichment of MMOx epibionts, after ^13^CH_4_ incubation. See *SI Appendix*, Table S2 for all ROI numbers and averages. (Scale bars: *A*, 50 µm. *D* and *E*, 5 µm. and *G*–*I*, 5 µm.)

The Del Mar *Sericosura* collected directly from the seafloor had an average tissue δ^13^C isotope value of −45 ± 3‰ (n = 6), even lighter than reported previously (12). To explore the possibility of carbon assimilation from methane or methanol into host tissues, live animals with intact bacterial communities were incubated onboard ship in the presence of ^13^C-labeled methane (100 atm%, ca. 0.4 mmol) or methanol (100 atm%, 250 or 500 uM), followed by bulk tissue isotope ratio mass spectrometry and single-cell resolved nanoscale secondary ion mass spectrometry (nanoSIMS; Figs. 4 and 5). Del Mar sea spiders incubated with ^13^C-methane (for 108 h) or ^13^C-methanol (for 171 to 181 h), had significantly enriched tissue δ^13^C values of +448 ± 139‰ (ANOVA *P* = 0.0006, F = 43.19) and +247 ± 120‰ (ANOVA *P* = 0.0038, F = 18.01), respectively (Fig. 4). The generation of ^13^C-enriched dissolved inorganic carbon (DIC) in the seawater surrounding ^13^C-methane- and ^13^C-methanol incubated sea spiders (newly formed bicarbonate = 161 µM and 42 µM, respectively) confirmed active conversion to carbon dioxide by sea spider-associated epibionts. Additionally, analysis of Del Mar seep amphipods similarly incubated with ^13^CH_4_ for 108 h showed no enrichment (δ^13^C value = −18 ± 1‰; n = 3), further supporting the specificity of ^13^C enrichment from methane by the sea spiders. Sea spiders exposed to ^13^C-bicarbonate had tissue δ^13^C values of −14 ± 3‰ (n = 2; Fig. 4), suggesting minimal incorporation of carbon from CO_2_.

Targeted single cell isotope imaging with nanoSIMS directly quantified ^13^C enrichment in sea spider tissues and epibiotic bacteria. Regions of interest (ROIs) were segmented for individual bacterial cells, along with zones of EPS and various sea spider tissues, including foregut and digestive diverticula found in their appendages (Fig. 5 and *SI Appendix*, Fig. S6). The digestive tissue of sea spiders exposed to ^13^C-methane was significantly enriched in ^13^C (0.014 ± 0.006), relative to those from the unlabeled ^12^C-methane control (ANOVA *P* = 0.0272, F = 5.09; Fig. 5 *C*–*E*, *SI Appendix*, Table S2). This ^13^C enrichment, reaching ~1.4× natural abundance within 5 d in the digestive tissue (Fig. 5*C*), indicated direct use of methane-derived carbon by the sea spiders. By comparison, the exoskeleton from these same spiders was not enriched (Fig. 5*C*), perhaps due to differences in material recalcitrance (chitin) and/or fast cell turnover time (digestive epithelial cells). Carbon incorporation from animals incubated with ^13^C-methanol could not be directly confirmed by nanoSIMS due to lack of suitable digestive tissue preparations. Bacterial epibionts, many of which appeared to be dividing (*SI Appendix*, Fig. S6*D*), were highly enriched in ^13^C when exposed to either ^13^C-labeled methane or ^13^C-methanol (up to 12× natural abundance; Fig. 5 *F*–*I* and *SI Appendix*, Table S2). During nanoSIMS analysis, we observed far fewer cells enriched in the ^13^C-methanol treatment than from methane, which was consistent with the 16S rRNA relative dominance of methanotrophic Methylomonadaceae relative to the low percentage of methanol-utilizing Methylophagaceae and Methylophilaceae (*SI Appendix*, Fig. S1). Even the EPS material surrounding the bacteria was enriched, especially in the ^13^C-methane treatment (by 9×; Fig. 5 *H* and *I*), thus implying possible methane-derived carbon sharing among the bacterial community on the sea spider surface.

## Discussion

Sea spiders in the genus *Sericosura* have only been documented from chemosynthetic habitats, including hydrothermal vents, methane seeps and whale falls (19, 20), suggesting a possible dependence on the high-energy chemicals that prevail at these sites. In this study, numerous individuals of three undescribed species of *Sericosura* sea spider were collected from two sites in California and a third site in Alaska. In all cases, MMOx bacteria comprised the majority of sea spider microbiomes. We found evidence for assimilation of methane-derived carbon by *Sericosura* via natural abundance δ^13^C isotopic measurements, and confirmed active incorporation using live animal incubations with ^13^C-labeled methane and nanoSIMS. Nearly all sea spider-associated MMOx bacterial cells assimilated carbon from either methane or methanol, but not CO_2_. Within five days, sea spider digestive tissues also showed significant incorporation of the ^13^CH_4_ label, a phenomenon that could only have occurred via consumption of methane-oxidizing bacteria.

The sea spider-associated microbiome consisted of multiple co-occurring methanotrophic and methylotrophic taxa, including three families known to associate with animals. For example, the Methylomonadaceae-MMG-2 are the primary symbionts of sponges, sabellid worms, and frenulates from deep-sea asphalt and methane seeps (3, 5, 6). The Methylophagaceae-MMG-3 occur as secondary symbionts in frenulates and the mytilid mussels *Bathymodiolus* and *Idas* spp. (3, 7–9) and the Methylophilaceae have been found as epibionts of the hydrothermal vent crab, *Shinkaia* (22). To date, however, no single animal species has been shown to host all three MMOx families. The population of bacteria involved in methane and methanol transformation with *Sericosura* is therefore uniquely diverse. In the current study, exclusive members of the Methylomonadaceae and Methylophagaceae taxa were especially enriched on the sea spiders, compared to authigenic carbonates, and the Methylophilaceae family was not recovered from any environmental sample. Most astonishing, sea spiders from off of southern California and Alaska share identical ASVs of both the Methylomonadaceae and Methylophagaceae. Thus, the *Sericosura* MMOx community appears to be specific and distinct from that of the seep habitat, despite residence on the external surfaces of the exoskeleton.

The methylotrophic members of the *Sericosura* microbiome could possibly colonize the host exoskeleton via transfer from parent to offspring either during creation of egg sacs or brooding, which can last as long as 20 d in some species (26). The exterior of the cohesive *Sericosura* egg sacs carried by the males hosted a similar MMOx community to the adults, providing a potential route for direct bacterial transmission between generations. Of the Del Mar seep specimens collected in situ, 50% were males, all of which were carrying eggs. This brooding prevalence is much higher than observed for other ammotheids (27), and could indicate enhanced fecundity at methane seeps. In an earlier description of *S. dissita* from hydrothermal vents in the northeast Pacific Ocean (28), it was stated that “a surprising number of males” were carrying egg clusters and that the only possible explanation for this high fecundity was that ample bacteria were available as a food source. Our findings add the possibility that these bacteria might have been associated with the animals themselves, as documented here for MMOx epibionts of seep *Sericosura*.

Methanotrophs and other methylotrophs often co-occur and share carbon directly, thereby driving microbial food webs in methane-rich environments (29, 30). Our nanoSIMS analysis provided evidence for both methane and methanol uptake by the microbial community and possible transfer of this carbon to other microbiome members via the EPS layer surrounding the epibiont aggregations. Direct carbon transfer can occur between members of the Methylomonadaceae, which produce methanol and other intermediates during methane oxidation, and both the Methylophilaceae and Methylophagaceae (23, 31). Transcriptomic analysis on MMOx cocultures demonstrated that the Methylophilaceae can actually induce their Methylomonadaceae neighbors to release methanol from the cell (32). Such interdependencies can occur within and upon animal tissues as well. The Methylophagaceae within the gills of bathymodioline mussels, for example, are thought to depend on the methanol produced by the metabolism of the primary methanotroph (a member of the Methylomonadaceae MMG-1 clade), thereby conferring enhanced nutritional flexibility to the animal host (7, 33). The diverse MMOx community associated with the sea spiders could take advantage of the ample flux of methane from the seeps in a similar cascade; the Methylomonadaceae could use methane as a primary energy and carbon source, releasing oxidation products which are then used by other microbial families. In this way, metabolic coordination among the *Sericosura*-associated MMOx bacteria may allow the sea spiders to exploit carbon derived from a range of C1 sources.

While tissue δ^13^C values < −40‰ are often observed in animals that rely on internal methane-oxidizing symbionts (1, 34), similar δ^13^C values can result from filtering, grazing, scraping, or deposit feeding on methanotrophic microbes in sediments, on carbonates, or from the water (35–37). In the case of *Sericosura*, microscopy revealed MMOx bacteria attached to the exoskeleton. These bacteria had intracytoplasmic stacked membranes characteristic of methanotrophs (25) and were arranged in regularly spaced exopolymer-covered aggregations, no more than a few cells thick. In many areas, the bacterial layers appeared disrupted, with cells removed entirely, leaving behind cellular footprints in the EPS scaffold. While the observed damage to the aggregations could have occurred during handling, these patterns are also consistent with active grazing by the sea spiders on the MMOx epibionts, and their associated EPS, similar to the way hairy yeti crabs farm and eat sulfur-cycling bacteria (22). In the case of the sea spiders, the animals could take advantage of their proboscis, which is exceptionally flexible in ammotheids (38). Further, *Sericosura* possess a set of triradial chitinous “lips” fringed with papillae (16), and three teeth (each ~10 μm in width; *SI Appendix*, Fig. S7) which could be used for grazing on the MMOx aggregations, either on other individuals or themselves. Active surface grazing has been observed for other ammotheids, including pressing their proboscis to their own bodies and consuming surface detritus and fouling bacteria (17, 39). *Sericosura* sea spiders are likely to rely on a combination of dietary sources, of which surface-bound MMOx communities are a large component. The wide variance in tissue δ^13^C values measured for *Sericosura* individuals (up to 15‰ in this study), supports the facultative nature of this symbiotic relationship.

## Conclusion

Sea spiders at hydrothermal vents and methane seeps are understudied. Little is known about how they fit into trophic food webs in these specialized environments, and thus far there have been no reports on the sea spider microbiome. Molecular analysis, microscopy, ^13^C-incubation experiments and nanoSIMS analysis revealed that ~50% of the microbes present on the exoskeleton were methanotrophs and methylotrophs, and that methane-derived carbon is incorporated into sea spider tissues. We therefore propose that at least three *Sericosura* species acquire organic carbon by grazing on abundant, aggregated epibiotic MMOx bacteria. Methane has been recognized as a potentially important source of carbon and energy for lake food webs, especially via grazing on sediment methanotrophs by detritivorous arthropods (37, 40). In the case of sea spiders, however, they appear to cultivate and consume the methane-oxidizing bacterial community from their bodies directly, rather than from abiotic surfaces.

*Sericosura* sea spiders now join the likes of other overlooked, yet abundant, small marine animals [e.g., serpulid tubeworms (6)] that take advantage of methane. A novel and unexpected animal–microbe dependency in an enigmatic animal group is particularly noteworthy in the Del Mar seep, given the relatively small seafloor habitat (~625 m^2^) in a well-trafficked area of the ocean. Dense populations of *Sericosura* have been observed at other seeps and vents, and this study suggests that they have the potential to form novel relationships with methane-cycling microbes. The finding that MMOx bacteria form unique associations with sea spiders at deep-sea methane seeps reveals a new biological conduit for methane input into ecosystems, and expands our understanding of C1 compound cycling in the ocean.

## Materials and Methods

### Sampling of Specimens.

Sea spiders of an undescribed species of *Sericosura* were collected from the Del Mar seep (32.90423 N/117.78223 W; 1,018 m depth) via carbonate rock collections and suction sampling during two expeditions (Fig. 1): in May 2021 aboard the R/V *Western Flyer* with ROV *Doc Ricketts* (operated by the Monterey Bay Aquarium Research Institute) and in June 2023 aboard the R/V *Atlantis* (AT50-12) with HOV *Alvin* (operated by the Woods Hole Oceanographic Institution). Dive DR1330 took place on May 20, 2021 (Fig. 1 *B* and *C*), and dives AD5193-AD5195 took place on July 17-19, 2023, respectively. A single specimen of a different species of *Sericosura*, also undescribed, was collected from the Palos Verdes seep (33.69936 N/ 118.37661 W; 379 m) on dive AD5200, ~100 km to the NW of the Del Mar seep. Two specimens of a different species of *Sericosura*, also undescribed, were collected from the Sanak seep (53.74865 N/ −162.58970 W; 2,020 m) on dive AD5278 and AD5279 (expedition AT50-24). The COI gene was amplified using previously published primers [LCO1490/ HCO21 (41)], and sequenced using Sanger sequencing, via Laragen Inc. (Culver City, CA). The COI sequences of the Del Mar and Sanak seep *Sericosura* species are available via GenBank accession numbers PP620304 and PQ663266 (*SI Appendix*, Fig. S8).

### Molecular Analyses of the Microbiome.

Specimens for molecular analysis were preserved within 2 h of collection in ~90% ethanol and stored at 4 °C. Total genomic DNA was extracted from whole specimens preserved in ethanol using the Qiagen DNeasy kit (Qiagen, Valencia, CA) according to the manufacturer’s instructions. Total DNA extracted was quantified via a Qubit 3.0 fluorometer using the dsDNA BR Assay Kit (Thermo Fisher Scientific). The V4-V5 hypervariable region of the 16S rRNA gene was PCR amplified using bacterial primers [515F: GTGYCAGCMGCCGCGGTAA and 806R: GGACTACHVGGGTWTCTAAT (42)] with Illumina adapters on the 5′ end (San Diego, CA). Each PCR product was secondarily barcoded with Illumina NexteraXT index v2 Primers that included unique 8-bp barcodes, with NEB Q5 Hot Start High-Fidelity Mix at an annealing temperature of 66 °C for 11 cycles. Barcoded products were purified using Millipore-Sigma (St. Louis, MO) MultiScreen Plate MSNU03010 with a vacuum manifold and quantified using the QuantIT PicoGreen dsDNA Assay Kit (Thermo Fisher Scientific) on a BioRad CFX96 Touch Real-Time PCR Detection System. Barcoded samples were combined in approximately equimolar amounts, purified again with Promega’s Wizard SV Gel and PCR Clean-up System (#A9281), and quantified again using the QuBit system. This sample was submitted to Laragen, Inc. (Culver City, CA) for 2× 250 bp paired-end analysis on the Illumina MiSeq platform with 20% PhiX addition. Raw reads were deposited in the NCBI archive under accession number PRJNA1104003.

Raw reads were processed as follows (43): CutAdapt v4.1 was used to remove the primer sequences, which allowed one error for every 10 bp in the primer sequence (44). FastQC v1.13 was used to quality control the raw sequence data and identify trim cutoffs for both the forward and reverse reads, ahead of pairing. Raw sequences were then processed with DADA2 [for initial quality trimming, error rate estimation, merging of read pairs, chimeric sequence removal and community data matrix construction (45)] and taxonomy was assigned to the processed ASVs (ASVs at 100% identity) using the SILVA database v138.1 (46). Nonmetric multidimensional scaling ordination, analysis of similarity and SIMPER analyses were performed in Primer-E, after square-root transforming the dataset and calculating Bray–Curtis similarities (47). Percent stacked bar charts were made with R packages dplyr (48) and tidyverse (49) in order to depict microbiome data [v4.3.1 (50)].

Additionally, an 823-bp fragment of the 16S rRNA gene was amplified using the primers 27F and MTC850R, the latter originally designed as a FISH probe targeting the MMG-2 group (4). Amplification products were sequenced using Sanger sequencing, via Laragen Inc., and submitted to GenBank (accession numbers PP346615-PP346620).

### Metagenomic Analysis.

Total genomic DNA was extracted from two specimens preserved in ethanol using the Qiagen DNeasy kit (Qiagen, Valencia, CA) according to the manufacturer’s instructions. Total DNA was pooled and quantified via a Qubit 3.0 fluorometer using the dsDNA BR Assay Kit (Thermo Fisher Scientific). Library construction was performed (on 0.48 μg DNA) and shotgun metagenomic sequencing was performed by Novogene Corporation, Inc. (Sacramento, CA) using the Illumina NovaSeq 6000 platform, resulting in ~263,438,828 reads (total output of raw data was 52.2G; Q30 ≥ 85%).

Quality control and primer and adapter removal was performed using bbduk (bbmap v38.87; sourceforge.net/projects/bbmap with the following parameters: minlen=100 mink=11 hdist=1 ktrim=r k = 23 tpe tbo ftr=149 qtrim=r trimq=10). The raw reads were assembled twice using megahit (51) (v1.2.9; with 21,29,39,59,79,99,119,141 kmers) or metaspades (52) (v3.15.2; with 21,29,39,59,79,99,119 kmers). 20 million reads were subsampled with reformat (bbmap) for the metaspades assembly. Metagenome-assisted genomes (MAGs) were binned and refined with metawrap (53) (v1.3.2), classified with GTDB-Tk (54) (r220) and annotated with METABOLIC (55) (v4.0). Based on checkM2 (56), bins with >50% completeness and <10% redundancy were kept. We focused our analysis on Methylococcales, Methylophilaceae and Methylophagaceae bins recovered from both assemblies. Phylogenetic trees were done in Anvi’o 7.1 (57), using the Bacteria_71 marker gene set. Genome size estimates were made by dividing the genome length by the checkM completion percentage. The carbonate MAGS used for comparison were sequenced and binned in a study on the same site and generated similarly (58). Based on manual Anvi’o inspection looking at the evenness of coverage of the selected sea spider MMOx MAGs, bins 1,4,5,7 (*SI Appendix*, Table S1) along with the raw reads, were uploaded to NCBI (PRJNA1171096). The complete set of MAGs is available on Figshare (10.6084/m9.figshare.27913776).

### Microscopy.

To assess the physical integration of microbes and *Sericosura* individuals, FISH microscopy and both TEM and SEM were performed. Specimens for FISH microscopy were initially preserved in 4% sucrose-buffered paraformaldehyde and kept at 4 °C for 24 to 48 h. These PFA-preserved specimens were rinsed with 2× PBS, transferred to 70% ethanol, and stored at −20 °C. Prior to embedding, specimens were treated for 1 wk with a 1:5 solution of hydrogen peroxide and ethanol to reduce autofluorescence. Samples were embedded in Steedman’s wax [one part cetyl alcohol: nine parts polyethylene glycol (400) distearate, mixed at 60 °C]. An ethanol:wax gradient of 3:1, 2:1, 1:1 and eventually 100% resin, was used to embed the samples (1 h each treatment). Embedded samples were sectioned using a Leica RM2125 microtome and placed on Superfrost Plus slides. Sections were dewaxed in 100% ethanol rinses. As a reference, some 5-μm sections were histologically examined via the Wright stain. To specifically target the dominant MMOx bacteria, a probe previously designed (4) to target the MMG-2 group of the Methylomonadaceae (MTC-850; 5′-ACGTTAGCTCCGCCACTA-3′) was labeled with the fluorochrome Cy5. A nonsense probe (59) (NonEub; 5′-ACTCCTACGGGAGGCAGC-3′), also labeled with Cy5, was used as a negative control (*SI Appendix*, Fig. S8). We also tested an additional probe (MTMC-701) designed (4) to target the Methylococcales more broadly, as well as probes specifically designed (24) for *Methylophilus* and related genera (MET-1217) and the Methylophagaceae MMG-3 group (MPH732). The samples were incubated in hybridization buffer containing 50 nM probe at 46 °C for 4 to 8 h, then in a hybridization buffer (0.9 M NaCl, 0.02 M Tris-HCl, 0.01% sodium dodecyl sulfate and 30% formamide) for 3 h, followed by a 15 min wash at 48 °C (0.1 M NaCl, 0.02 M Tris-HCl, 0.01% sodium dodecyl sulfate, 5 mM EDTA). Sections were counterstained with DAPI (5 mg/mL) for 3 min, rinsed and mounted in VECTASHIELD® Vibrance™ Antifade Mounting Medium (Vector Laboratories, Newark, CA). Tissues were examined by epifluorescence microscopy using a Nikon E80i epifluorescence microscope with a Nikon DS-Qi1Mc high- sensitivity monochrome digital camera.

Specimens for SEM were placed directly into 1% osmium tetroxide for 30 to 60 min, then rinsed in distilled water for several days, and stored in distilled water (all steps at 4 °C). Specimens were then rehydrated in a graded ethanol series (30%, 50%, 75%, 95% and 100%, 15 min each) and dried using a samdri-PVT-3D critical point dryer (Tousimis Research Corp, Rockville, MD). Specimens were then mounted via adhesive copper tape (Ted Pella 16072) and PELCO conductive carbon glue (Ted Pella 16050), gold-coated using a Pelco SC-4 4000 sputter coater and visualized using either a Zeiss EVO 10 Scanning Electron Microscope (for bacterial morphology) or a Phenom Pro’X G6 Desktop SEM (for spider morphology).

Specimens for TEM were initially preserved in 3% glutaraldehyde buffered with 0.1 M phosphate and 0.3 M sucrose (pH 7.8). The fixed samples were dehydrated in a graded series of cold acetone, and embedded in epoxy resin (Oken Epok 812; Oken-shoji, Tokyo, Japan). Ultrathin sections (60 to 80 nm) were cut with a diamond knife, transferred to copper grids (50 mesh) that had been coated with Formvar membrane, stained with uranyl acetate and lead citrate and observed with a Hitachi HT7700 TEM by Creative Bioarray (Shirley, NY).

### Shipboard Isotope Labeling Experiments and Analysis.

Specimens collected for stable isotope analyses were initially rinsed in Milli-Q water and frozen onboard ship at −20 °C, then in the laboratory were oven-dried at 60 °C overnight, weighed, and acidified with 1 N phosphoric acid to remove inorganic carbon. δ^13^C measurements were made on 0.2 to 2.5 mg dry-weight samples combusted using a PDZ Europa ANCA-GSL elemental analyzer interfaced to a continuous flow PDZ Europa 20-20 isotope ratio mass spectrometer at the Stable Isotope Facility at University of California, Davis. Isotopic values were generally consistent among all major body parts, including proboscis (which bears the mouth), trunk and legs.

To assess interactions between MMOx bacteria and the sea spiders, short-term incubation experiments with ^13^C-labeled methane and ^13^C-labeled methanol were conducted at sea. Sea spiders not exposed to any carbon substrate were included as a control. Sea spiders exposed to ^13^C-labeled bicarbonate (100 atm%, 2 mM addition) were included to test for other forms of chemoautotrophy (e.g., sulfide oxidation coupled to carbon fixation). All treatments were incubated at 10 °C in 60 mL serum bottles filled with 0.2-mm filtered bottom seawater from the collection site. Experimental incubations were augmented with either ^13^C-labeled methanol (250 or 500 uM final concentration, for 171 to 181 h) or ^13^C-labeled methane (100 atm%, 0.4 mmol, for 108 h). At these concentrations, methane is saturated and dissolved at conditions close to those found in situ at methane seeps. For downstream nanoSIMS analysis, 100% labeling strength was used in order to enrich heavy isotopes into biomass during short shipboard incubations. In addition, water samples (~200 µL) for DIC measurements were filtered through a 0.2-mm polyethersulfone filter into helium (He)-flushed, 12 mL Exetainer vials (Labco Ltd., Lampeter, UK), following the addition of 200 µL of ~40% phosphoric acid. At the end of each incubation, sea spiders, amphipods or limpets (the latter two as comparison animals) were rinsed in Milli-Q water, and frozen at −20 °C until analysis, as described above for natural specimens. The overall precision of this technique is estimated to be > ±0.2‰, based on multiple analyses of independent standards as samples.

Newly formed DIC measurements were made from 0.25 to 0.5 mL of 0.2 μm filtered liquid from incubations, initially added to pre-Helium-flushed vials containing 200 µL of ~40% phosphoric acid onboard the ship. Measurement of newly formed DIC via ^13^CH_4_ or ^13^C-methanol conversion was measured and quantified using gas chromatography isotope ratio mass spectrometry (GasBench II; Thermo Scientific) (60).

### NanoSIMS.

^13^C-exposed sea spiders were preserved in 4% sucrose-buffered paraformaldehyde and kept at 4 °C for 24 to 48 h, rinsed with 2× PBS, transferred to 70% ethanol, and stored at −20 °C. Specimens were embedded in Steedman’s wax (via an ethanol:wax gradient of 3:1, 2:1, 1:1, and eventually 100% resin; 1 h each treatment). Semithin sections between 2 to 3 μm were cut dry using a Leica RM2125 microtome with a metal blade. Sections were deposited in ∼20 μL deionized water droplets on poly-L-lysine-coated wells of glass slides (Tekdon Incorporated). Regions with bacterial aggregations on the exoskeleton surface were mapped using transilluminated light and etched on a laser microdissection system (Leica LMD 7000), with reference to fluorescence-microscopy (using MTC850) on neighboring sections, as described above. Slides were then scored with a diamond scribe, broken and filed to fit into the nanoSIMS sample holder (~1 cm diameter) and sputter-coated with a 20-nm thick layer of gold using a Cressington sputter coater. NanoSIMS analysis was carried out using a Cameca NanoSIMS 50L housed in Caltech’s Microanalysis Center. Etched areas were then identified using the nanoSIMS CCD camera. Samples were presputtered with a 100-pA primary Cs+ ion beam (aperture diaphragm D1 = 1) until the 12C15N– ion counts stabilized. Data were then collected from bacteria and sea spider tissues using a 7-pA primary Cs+ ion beam (D1-2), at 512 × 512 pixel resolution, and an average raster size of 20 to 30 μm^2^. Masses (^12^C-, ^13^C-, ^12^C^14^N-, ^15^N^12^C-) were collected in parallel using electron multipliers with a dwell time of 12 to 48 ms/pixel. Mass calibration was performed every ∼1 h for all masses. NanoSIMS.im format files were initially processed with the look@nanoSIMS package (61). ROIs were segmented manually on a tablet using the ^12^C- raw frame as the template (*SI Appendix*, Fig. S6). Patches for bacterial EPS were segmented with contours several pixels away from bacterial cells. For exoskeleton tissue, regions without highly enriched bacterial cells nearby were segmented and measured to exclude any possibility of cross contamination during microtome sectioning and nanoSIMS sputtering. All subsequent data processing and analysis was done in Matlab (v. 2020b) and the final results are shown as the enrichment ratio (^13^C/^12^C).

## Supplementary Material

Appendix 01 (PDF)

## Data Availability

The raw Illumina 16S rRNA barcode sequences and metadata collected in this study are available from the NCBI Small Read Archive (PRJNA1104003) (62). Methylococcales-specific 16S rRNA sequences generated in this study are available via GenBank accession numbers PP346615–PP346620 (63–68). The COI gene sequences of the Del Mar and Sanak seep *Sericosura* are available via GenBank accession numbers PP620304 (69) and PQ663266 (70). MAGs for select MMOx bacteria (bins 1,4,5,7; *SI Appendix*, Table S1) along with associated SRA metadata are available via NCBI (PRJNA1171096) (71). The complete set of MAGs is available on Figshare (10.6084/m9.figshare.27913776) (72). Animal images and specimens were vouchered (Del Mar species catalog nos. C14441 and W1012, and Sanak species catalog nos. W10078 and W10405) for long-term archiving into the Benthic Invertebrate Collection at Scripps Institution of Oceanography (https://sioapps.ucsd.edu/collections/bi/) (73).
