# Supplementary material for: Methane-powered sea spiders: Diverse, epibiotic methanotrophs serve as a source of nutrition for deep-sea methane seep Sericosura
Source: Proc Natl Acad Sci U S A. 2025 Jun 16;122(26):e2501422122. doi: 10.1073/pnas.2501422122 (PMC12232434; doi:10.1073/pnas.2501422122)
Supplement: Supplementary file 1 — Appendix 01 (PDF) [file pnas.2501422122.sapp.pdf]

Supporting Information Appendix

Methane-powered sea spiders: Diverse, epibiotic methanotrophs serve as a source of nutrition for deep-sea methane seep *Sericosura*

Bianca Dal Bó[1], Yongzhao Guo[2], Magdalena J. Mayr[2], Olivia S. Pereira[3], Lisa A. Levin[3], Victoria J. Orphan[2], Shana K. Goffredi[1*]

Table S1. Metagenome-assembled genome (MAG) highlights for methanotrophic and methylotrophic (MMOx) bacteria associated with *Sericosura* sea spiders.

Table S2. Summary of ^{13}C enrichment in bacteria and *Sericosura* sea spider tissues at single cell level from nanoSIMS analysis.

Figure S1. Analysis of the *Sericosura* microbiome, both adults and egg sacs, based on the 16S rRNA gene amplicon sequencing.

Figure S2. Phylogenetic relationships among the 16S rRNA amplicon sequence variants (ASVs) attributed to methanotrophic and methylotrophic bacteria associated with *Sericosura* sea spiders.

Figure S3. Overview of selected genes of interest within MAGs from abundant Methylococcales, Methylophagaceae, and Methylophilaceae.

Figure S4. Phylogenetic relationships among the genes associated with methanotrophic and methylotrophic metabolism, recovered from environmental metagenomics.

Figure S5. Microscopy of *Sericosura* egg sacs, and associated bacteria.

Figure S6. The nanoSIMS measurements collected from a cross section of spider pedipalp tissue.

Figure S7. Imaging of the mouth-bearing proboscis of *Sericosura* sp. nov. from the Del Mar seep.

Figure S8. Sea spider phylogeny, based on mitochondrial cytochrome c oxidase, showing the placement of new species discovered during this study.

Figure S9. Fluorescence microscopy of *Sericosura* egg sacs, and associated bacteria, comparing positive to negative results.

Table S1. Metagenome-assembled genome (MAG) highlights for methanotrophic and methylotrophic (MMOx) bacteria associated with *Sericosura* sea spiders.

Taxon (GTDB)	Bin #	genome size (Mb)[1]	CheckM2 Completeness	CheckM2 Contamination	coverM (avg)	MMOx Genes[4]
Methylococcales						
Methylomonadaceae	01	3518624	99.9[2]	0.0	5.7	pmoCAB, MDH
Methylomonadaceae	03	3325478	86.8[3]	2.4	0.8	pmoCAB, MDH
Methyloprofundus sp.	06	5666188	70.0[2]	2.9	0.5	pmoCB, MDH
Methylococcales no id	02	3061509	92.0[3]	2.6	4.2	pmoBA
Nitrosococcales						
Methylophagaceae	04	2324860	79.9[2]	0.4	0.8	pmoCAB, MDH
Methylophagaceae	07	2087069	96.6[3]	4.0	0.3	MDH
Burkholderiales						
Methylophilaceae	05	1977021	86.3[2]	0.2	0.6	MDH

[1]Genome sizes averaged from combined metaspades and megahit assemblies
[2]from metaspades assembly, if most complete
[3]from megahit assembly, if most complete
[4]from both assemblies; pmoCAB = subunits of particulate methane monooxygenase; MDH = coding for the large subunit of methanol dehydrogenase

Table S2. Summary of ^{13}C enrichment in bacteria and *Sericosura* sea spider tissues at the single cell level from nanoSIMS analysis.

	Bacterial cells_CH_4	Bacterial cells_MeOH	Bacterial cells_control
$^{13}C/(^{13}C+^{12}C)$	0.128 ± 0.120	0.078 ± 0.047	0.010 ± 0.001
ROI number analyzed	1,992	106	341
	Bacteria EPS_CH_4	Bacteria EPS_MeOH	Bacterial EPS_control
$^{13}C/(^{13}C+^{12}C)$	0.103 ± 0.084	0.041 ± 0.029	0.010 ± 0.001
ROI number analyzed	173	15	37
	Digestive tissue_CH_4	Exoskeleton_CH_4	Tissue_control
$^{13}C/(^{13}C+^{12}C)$	0.014 ± 0.006	0.011 ± 0.001	0.010 ± 0.001
ROI number analyzed	47	6	10

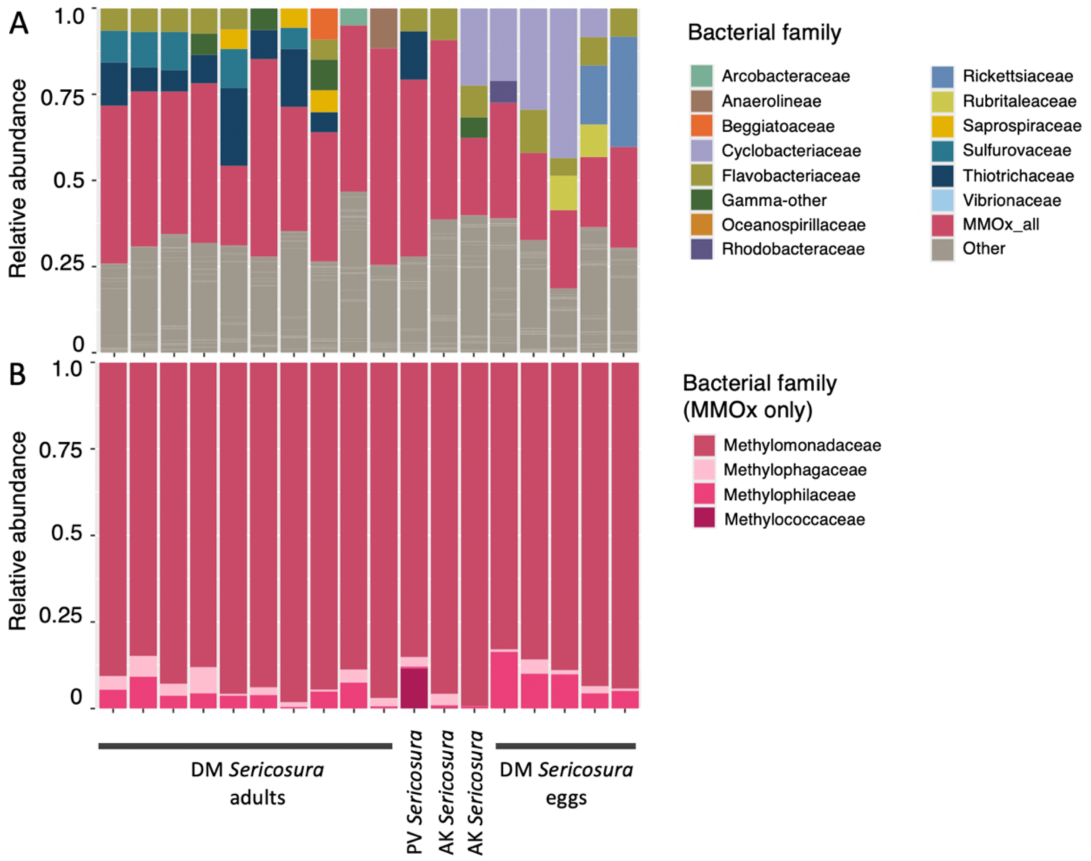

Figure S1. Analysis of the *Sericosura* microbiome, both adults and egg sacs, based on the 16S rRNA gene amplicon sequencing. Nomenclature follows the Genomic Taxonomy Database (University of Queensland, Australia). Stacked bar plots show the relative abundance of bacterial families. A) Showing the full bacterial community, with all 4 methanotrophic and methylotrophic families (MMOx_all; red tones) combined and bacterial families with abundances <5% in a given specimen combined in the "other" category, shown in grey. The egg sac microbial assemblage was distinct from the adults (ANOSIM R = 0.70, p = 0.01), primarily due to predominance of the Cyclobacteriaceae. B) Showing only the MMOx families (out of 100%). In addition to MMOx families, metagenomic sequencing recovered partial genomes for the Thiotrichaceae, Flavobacteriaceae and Saprospiraceae, among others (https://figshare.com/s/b6122865b0067a0b695b). DM, Del Mar methane seep, CA. PV, Palos Verdes seep, CA. AK, Sanak seep, Alaska.

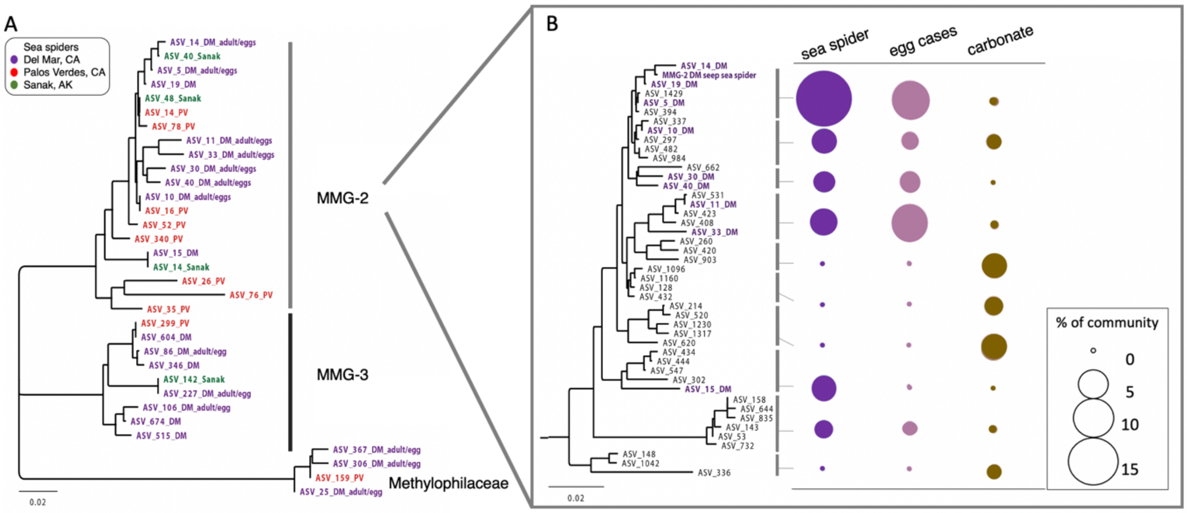

Figure S2. Phylogenetic relationships among the 16S rRNA amplicon sequence variants (ASVs) attributed to methanotrophic and methylotrophic bacteria associated with *Sericosura* sea spiders. A) 16S rRNA ASVs from all 3 *Sericosura* species examined in this study, color-coded by site, including both the adults and eggs from the Del Mar specimens. B) Distribution of 16S rRNA ASVs of just the Methylomonadaceae MMG-2 group in the Del Mar *Sericosura* sea spider microbiome (n = 10), compared to those recovered from adult male egg cases (n = 5) and the exterior of carbonates collected nearby (n = 2). Circle size indicates the % of the total bacterial community. At left in panel B is a phylogenetic tree showing the relationships among the ASVs, based on a neighbor-joining tree and Tamura-Nei model. All sea spider-associated ASVs are shown in bold-purple. The 'MMG2-DM seep sea spider' sequence corresponds to that shown in Figure 2A, recovered via direct 16S rRNA sequencing.

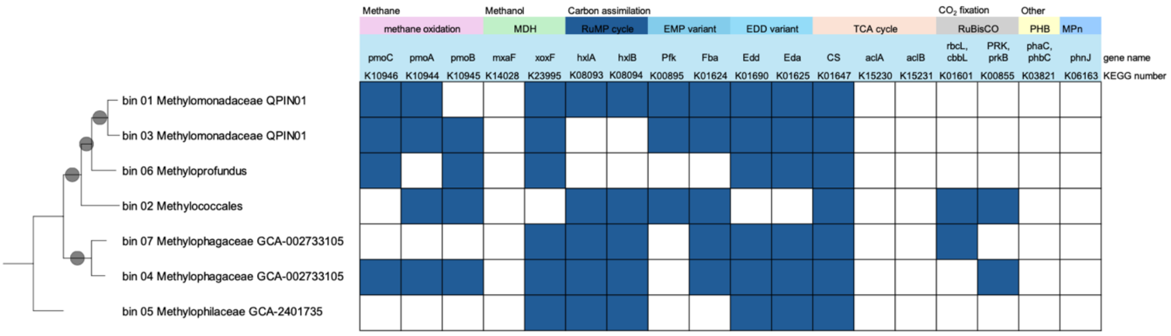

Figure S3: Overview of selected genes of interest within metagenome-assembled genomes (MAGs) from abundant Methylococcales, Methylophagaceae and Methylophilaceae. Replicate MAGs were reconstructed using MEGAHIT or metaSPAdes assemblies, if one or both encodes the respective gene the box is coloured blue. The completeness and contamination of the higher quality bin (CheckM2) are shown in Table S1. The phylogenomic tree on the left was constructed in Anvi'o and visualized using iTOL. All displayed bootstrap values (circles) are significant, with a value of 1. Note that MAGs are inherently incomplete. Abbreviations: methanol dehydrogenase (MDH), ribulose monophosphate pathway (RuMP), Embden-Meyerhof-Parnas pathway (EMP), Entner-Doudoroff pathway (EDD), ribulose-1,5-bisphosphate carboxylase (RuBisCo), polyhydroxybutyrate (PHB), methylphosphonate cleavage (MPn). Gene names: Methane monooxygenase subunits (pmoCAB), methanol dehydrogenase subunit (mxaF), lanthanide-dependent methanol dehydrogenase (xoxF), 3-hexulose-6-phosphate synthase (hxlA), 6-phospho-3-hexuloisomerase (hxlB), diphosphate-dependent phosphofructokinase (Pfk), fructose-bisphosphate aldolase (Fba), phosphogluconate dehydratase (Edd), 2-dehydro-3-deoxyphosphogluconate aldolase (Eda), citrate synthase (CS), ATP-citrate lyase subunits (aclA, aclB), ribulose-bisphosphate carboxylase large chain (rbcL, cbbL), phosphoribulokinase (PRK, prkB), poly[(R)-3-hydroxyalkanoate] polymerase subunit (PhaC, phbC), alpha-D-ribose 1-methylphosphonate 5-phosphate C-P lyase (phnJ).

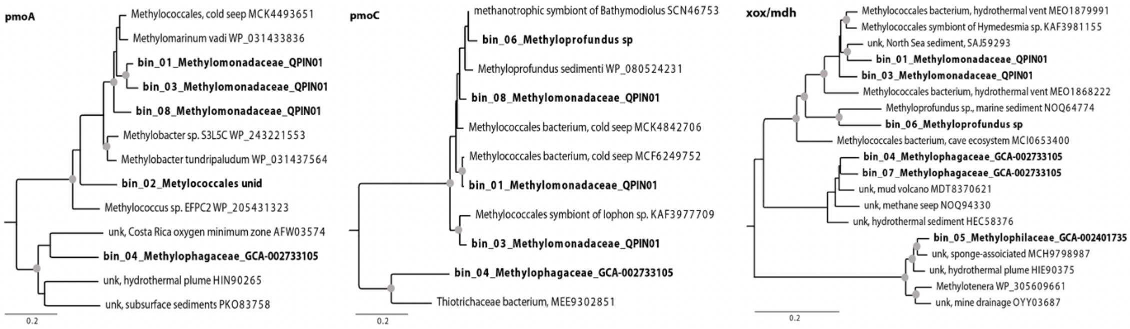

Figure S4: Phylogenetic relationships among the genes associated with methanotrophic and methylotrophic metabolism, recovered from environmental metagenomics. The particulate methane monooxygenase genes (pmoA and C) and methanol dehydrogenase gene (e.g. xox; commonly characterized as a methanol/ethanol family PQQ-dependent dehydrogenase) are shown. Trees are based on protein alignments (neighbor-joining, Jukes-Cantor model), using Geneious Prime 2021.2.2. Additional sequences from environmental and cultured relatives were obtained from GenBank. Circles indicate bootstraps above >0.7. The pmoCAB identified in Methylophagaceae (bin_04) is divergent from previously characterized methanotroph sequences. A similar pmoCAB was found on carbonate surfaces in reference 58. Currently, the most similar pmoC in NCBI (68% identity) belongs to an uncultivated Thiotrichaceae MAG with an unknown function.

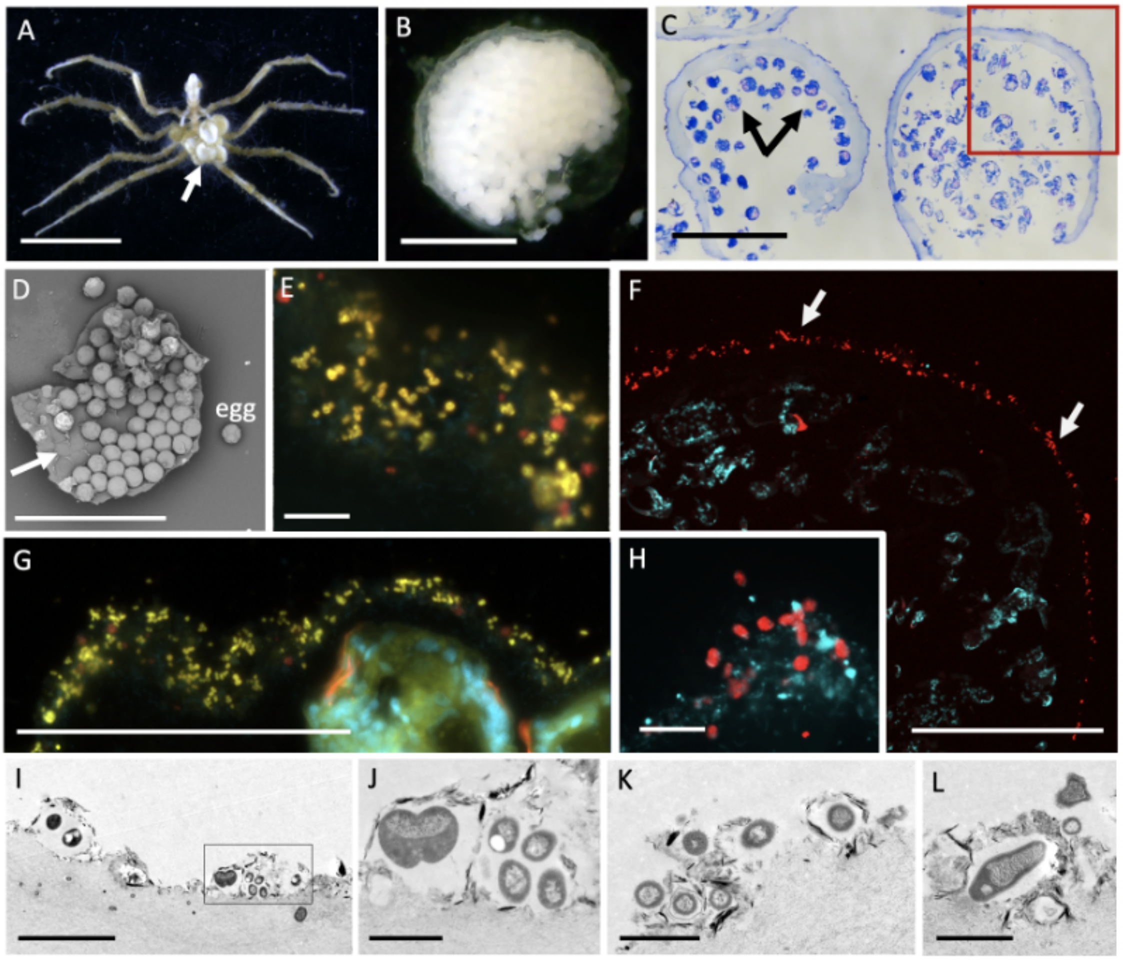

Figure S5. Microscopy of *Sericosura* egg sacs, and associated bacteria. A) Male *Sericosura* specimen carrying 10 egg sacs (arrow). B) Image of an egg sac after removal from the male ovigers. C) Light microscopy of a Wright-stained cross section (5-µm thick) through 2 egg sacs embedded in Steedman's resin, with eggs inside (arrow). The eggs in the righthand egg sac are in a more advanced stage of development than those on the left. Box denotes region in E. D) Scanning electron microscopy (SEM) showing numerous eggs within a dissected egg case. E) Fluorescence in situ hybridization (FISH) of the external surface of an egg sac, revealing numerous MMOx cells, hybridized with a probe designed to broadly target the Methylococcales (MTMC701, labeled with Cy5) and the Methylomonadaceae more specifically (MTC850 labeled with Cy3, Ruff et al. 2013). An overlap between probes is shown in yellow, while the MTMC801 probe alone is shown in red. F) FISH image of the external surface of an egg sac, revealing MMOx cells (in red, arrows, hybridized with the MTC850 labeled with Cy5, in red), and the developing spider brood within (stained with DAPI, in cyan). Red signal inside of the egg sacs was artificial and also observed in the negative control. G). Same probes as in E, showing the Methylococcales and Methylomonadacea simultaneously, in yellow. H) FISH image showing MMOx cells targeted by the Methylococcales probe (MTMC701, labeled with Cy5), and other non-Methylococcales cells in cyan, stained with DAPI. I-L). Transmission electron microscopy (TEM) of various bacterial cells associated with the surface of the egg sacs. Box in I denotes region in J. Scale bars: A, 5 mm. B-D, 500 µm. E, 10 µm. F, 250 µm. G, 100 µm. H, 10 µm. I, 5 µm. J-L, 1 µm.

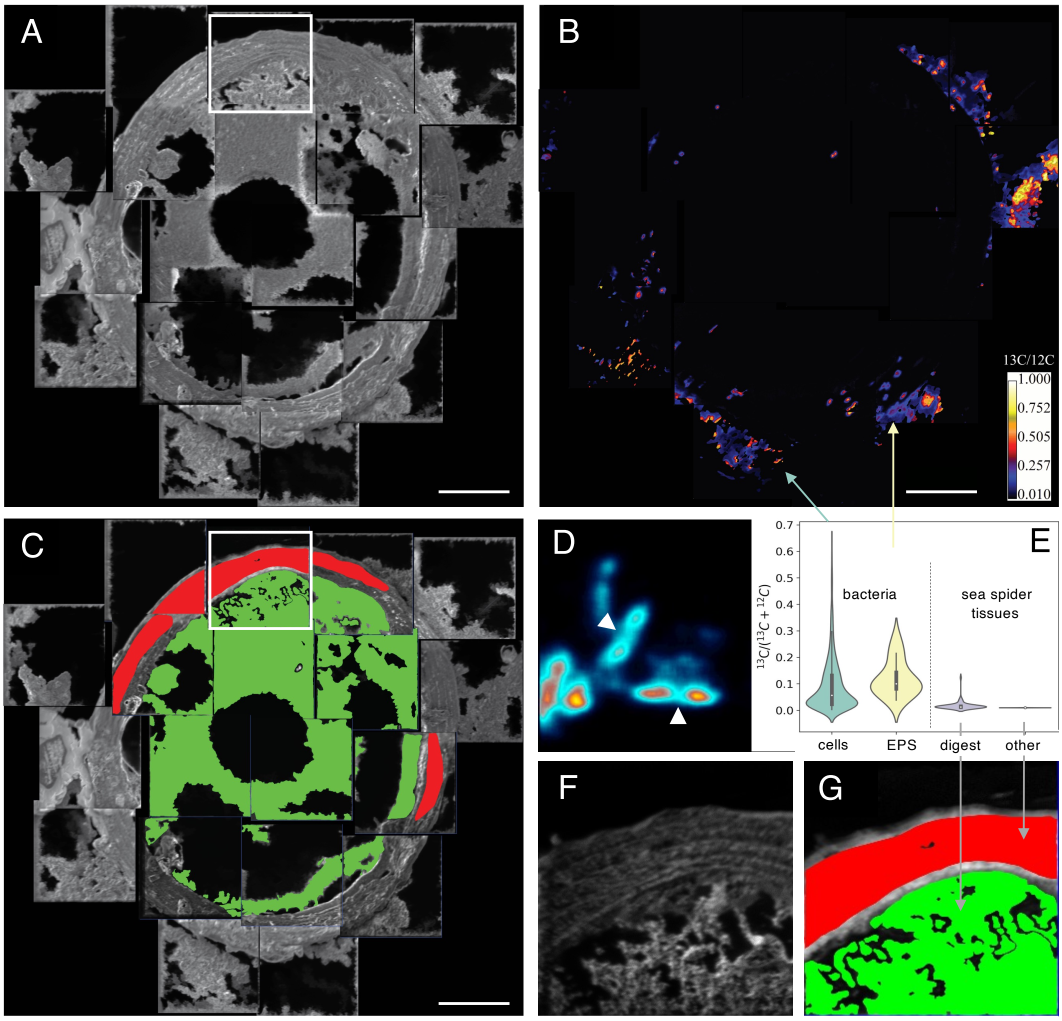

Figure S6. The nanoSIMS measurements collected from a cross section of sea spider pedipalp tissue. A) The mosaic 12C− ion mass images cover the whole pedipalp region of interest. B) nanoSIMS image revealing ^{13}C enrichment after $^{13}CH_4$ incubation. Warmer colors indicate higher enrichment. C) The mosaic images of 13C−/12C− across the pedipalp. The ratio images were illustrated by Limage PV-WAVE (v9.00). Segmentations were identified as digestive tissue (green) and exoskeleton muscle (red) within the pedipalp tissue. D) Dividing epibiotic bacteria (arrowheads), as revealed by the 12C− ion mass image. E) Violin plot of nanoSIMS data, showing ^{13}C enrichment levels in sea spider-associated bacterial epibiont cells, EPS layer, digestive tissue, and other tissue (in this case exoskeleton) when exposed to ^{13}C-methane. Arrows indicate regions used for analysis in G. F-G) Higher magnification of regions shown in A/C (boxes), which correspond to the data show in E. Scale bars: A-C, 20 µm.

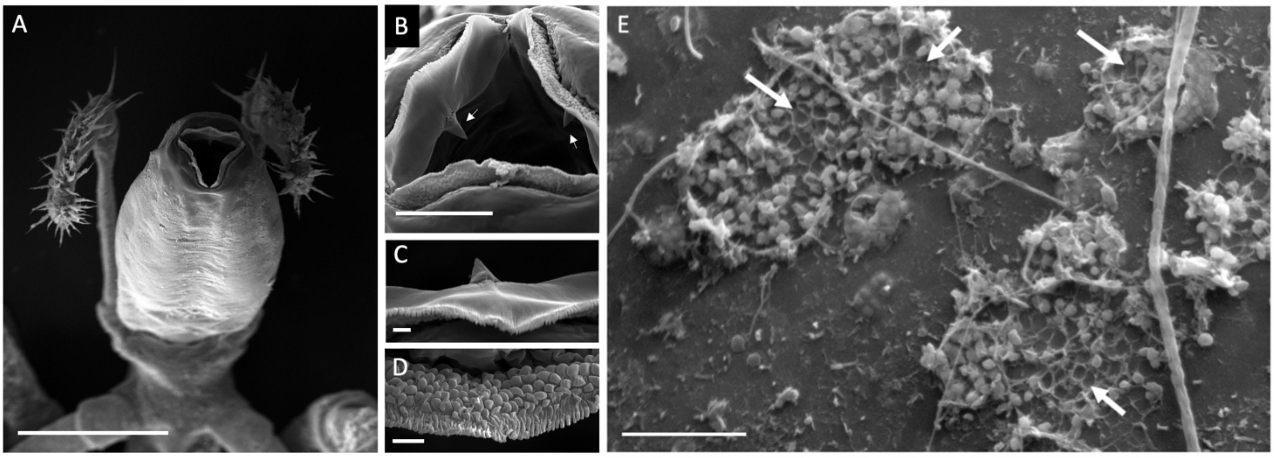

Figure S7: Imaging of the mouth-bearing proboscis of *Sericosura* sp. nov. from the Del Mar seep. A) Scanning electron microscopy (SEM) of the ventral surface of the proboscis and pedipalps. B) The triangular mouth opening, showing 2 of the 3 internal teeth (arrows). C) Close-up of one of the teeth. D) Close-up of calcified lip margin. E) SEM image of cells clustered together in several aggregates, some of which were intact, and others where bacteria appeared removed or were missing (arrows; see also Figure 3H). Scale bars: A, 500 µm. B, 100 µm. C-E, 10 µm.

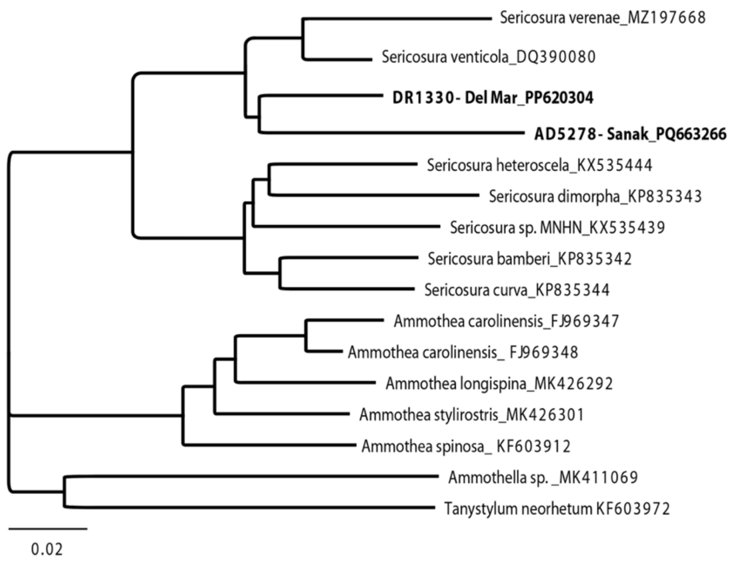

Figure S8. Sea spider phylogeny, based on the mitochondrial cytochrome c oxidase gene, showing the placement of new species discovered during this study (taxa in bold). The tree is based on a neighbor-joining Tamura-Nei model, aligned using Geneious Prime 2021.2.2. Additional sequences were obtained from GenBank.

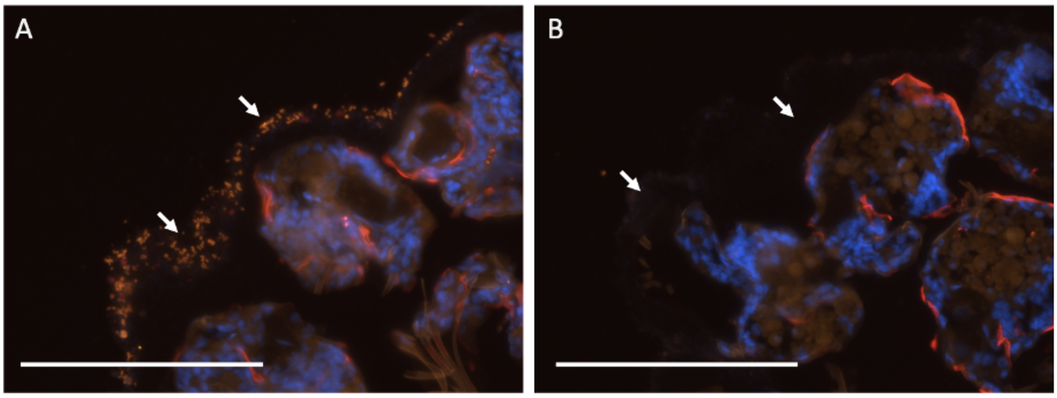

Figure S9. Fluorescence microscopy of *Sericosura* egg sacs, and associated bacteria, comparing positive to negative results. A) FISH image of a *Sericosura* egg case, showing a layer of MMOx bacteria (arrows) targeted by a combination of the Methylococcales probe (MTMC701 labeled with Cy5) and Methylomonadacea probe (MTC850 labeled with Cy3). An overlap between probes is shown in light orange. B) The same *Sericosura* egg case, hybridized with a probe designed not to target bacteria (NonEub; 5′-ACTCCTACGGGAGGCAGC-3′), also labeled with Cy5. Some autofluorescence can be seen in both images as an orange outline of the developing embryos. Scale bars: A-B, 100 μm.
